# Notices of New Works

**Published:** 1836-07-01

**Authors:** 


### Notices of some New Works.

1. THE CYCLOPÆDIA OF ANATOMY AND PHYSIOLOGY. Edited by ROBERT TODD, M.B. &c. &c. &c. Parts V. and VI.

THIS meritorious work proceeds with regularity, and, we hope, with success. When completed, its volumes will be valuable to every surgeon, for reference and for instruction.

The Fifth Part commences with the conclusion of an article on the Morbid Conditions of the Blood. The Sixth Part terminates with the commencement of a description of the cilia in the infusoria, polypi, acalephæ, &c. The intermediate articles are on the normal and morbid anatomy of bone—the brachial artery—the bursæ—the carnivora—the carotid artery—cavity—cellular tissue—cephalopoda—cerumen—cetacea—cheiroptera—chyliferous system—cicatrix. The descriptions are in general very good—the engravings numerous—the style of the Fasciculi very creditable to all parties.

It would be ridiculous to offer an analysis, and it would be difficult to present a review of an elementary production like this. Yet when we use the term elementary, we are sensible that we do injustice to many of the contributors, who have thrown together information that could scarcely be collected by the majority of readers, and added the results of their own reflection and observation. We cannot pretend to have perused all the closely printed pages, and therefore, after uttering a general and a well-deserved encomium, we must content ourselves with a desultory glance at one or two of the more prominent articles.

Mr. Owen, the able conservator of the Museum of the College of Surgeons, has communicated an elaborate and an excellent memoir on the cephalopoda. Perhaps the following extract on the "ink bag" of the cephalopoda, may display in some degree Mr. Owen's style. His varied and extensive information cannot be so easily exhibited.

The apparatus for secreting an inky fluid is wanting in the nautilus, which is defended by its strong shell.

"The ink-bag varies in its relative position in different *Dibranchiata*: in the Cuttle-fish it is situated near the bottom of the pallial sac, in front of the testicle or ovary. In the Calamary it is raised close to the termination of the intestine; we have found it similarly situated in the Argonauta, Sepioteuthis, and Rossia. In the Octopus it is buried in the substance of the liver, a small part only of its parietes appearing on the anterior surface of that gland, from which its duct is continued forwards to terminate in this genus immediately behind the anus.

From this connection of the ink-bag with the liver in the Poulp, Monro was led to suspect it to be the gall-bladder. What its real nature may be still remains doubtful; De Blainville and Jacobson regard it as a rudimental urinary apparatus: Sir Everard Home compares it to the secreting sac which opens into the rectum in Rays and Sharks, and this we consider to be the true homology of the ink-bag. It is interesting, indeed, to observe that corresponding anal glandular cavities in the Mammalia are in many instances modified to serve by the *odour* of their secretion as a means of defence, just as the part in question operates in the Cephalopods by reason of the *colour* of the ejected fluid.

When the ink-bag is laid open and well cleansed of its contents, its inner surface is seen to be composed of a fine cellular or spongy glandular substance: its exterior coat is of a tough white fibrous texture, and its outer surface commonly exhibits a peculiar glistening or silvery character.

The ink-bag probably attains its largest proportional size in the genus *Sepiola*, where it presents a trilobate form. It is of an oblong pyriform shape in *Sepia, Sepioteuthis*, and *Loligo*. It is relatively larger in *Sepia* than in *Octopus*, and the quantity of water which its contents will discolour is very surprising: it behoves the anatomist, therefore, to be very careful not to puncture this part during the dissection of a Cephalopod.

In the living Cephalopods the inky fluid is secreted with amazing rapidity; we have seen an Octopus, which had previously discoloured the water for a considerable extent around it, immediately after its capture continuing its black ejections several times in quick succession, and ultimately expelling in convulsive jets a colourless fluid, when the powers of secreting the black pigment were exhausted.

In every species of Cephalopod which possesses this organ, the tint of the secretion corresponds, more or less, with the coloured spots on the integument. The Italian pigment, called 'Sepia,' and the Chinese one, commonly called 'Indian Ink,' both of which are the inspissated contents of the organ above described, afford examples of different shades of this singular secretion.

If the Cephalopods are enabled thus to conceal themselves during the day, they have also the power, by means of another secretion, to render themselves conspicuous by night by means of a phosphorescent exhalation."

We think there can be little doubt that the opinion advanced by Sir E. Home, is the most philosophic one, —that the ink-bag in the cephalopods is analogous to the secreting organ near the anus in other creatures; and that it serves as an apparatus of defence. The illustrations of the article on cephalopoda, are profusely lavished on the reader. Mr. Owen is a great acquisition as a contributor to the work.

We may notice some remarks on the properties of a cicatrix by Mr. A. Dodd.

The difference between newly-formed skin and the old skin are thus enumerated.

1. The new skin, as is well known, occupies a smaller space than the old; whence the familiar contraction of cicatrices. Of course, this contraction is proportioned to the laxity of the subjacent cellular tissue, and surrounding parts.

2. The texture of the cicatrix is frequently harder and thicker than the natural skin. This circumstance varies considerably, but we believe this variation will be found to bear a pretty exact relation to the degree of contraction, to the length of time occupied in the cure, and to the irritation to which the ulcer was subjected in the process of healing. When these have been considerable, the hardness is correspondingly great, while, if the cure has been expeditious and the part been kept extended and irritation avoided, the cicatrix remains soft, thin, and pliable, a point of great importance in practice as applied to the healing of burns. 3. The colour of the new skin is different from the natural parts. This arises from the want of rete mucosum, which is not regenerated till long after the other tissues, and sometimes not at all. For this reason a cicatrix in a Black is as white as that in an European; but after a considerable lapse of time, this structure is sometimes formed anew, and in some instances becomes even of a darker colour than before. 4. The surface is perfectly dry from the want of exhalent pores, which are never found to be restored even in the oldest cicatrices. Indeed, in cases where the chorion has not been destroyed through its entire thickness, the loss of substance reaching only through its outer layers, these pores are generally obliterated, and the important exhalent function of the skin is annihilated; and even when the injury has extended only through the external vascular structure of the skin, as is the case in the healing of a blister which has been long inflamed, we have observed a drier state of the parts, and more polished than the surrounding skin which had not been injured. From this peculiarity in the cicatrix, when the whole body is bathed in sweat these parts are dry and polished. This state of dryness, however, partly results from another anatomical deficiency, namely, of the perspiratory glands, which are destroyed in cases where the entire integument has been injured, and these are of course never regenerated. 5. The new tissue contains no hairs, and if, after superficial wounds, a few scattered hairs appear on the surface, they are feeble and white. 6. After the healing of a large ulcer of long standing, the new surface is sometimes much lower than the surrounding skin. 7. The elasticity of the cellular tissue under the new skin is diminished. This is seen in œdema and in emphysema. The restoration of the nervous and vascular functions in the cicatrix, doubtless varies in amount, in different cases. But a cicatrix is after a time less vascular than the skin was previously, as may be seen by its white colour, and imperfect injection.

"On the temperature of the cicatrix we have not made sufficient observations to generalize, but we have found that the actual temperature of the bridle from a burn, while it retains its hardness, is several degrees above that of the healthy skin, while the power of retaining its temperature, or of resisting the extremes of heat and cold, is much inferior in the cicatrix to that which the healthy skin possesses, although the actual temperature, *under ordinary circumstances,* is the same as the surrounding skin. Almost every traveller to the Poles or to the Tropics mentions the liability of old ulcers that had been healed, to announce the extremes of temperature by pain and inflammation."

In picking out the preceding passages, we are sensible that we may not have done justice to the work, nor indeed to the very authors we have quoted. But we can conscientiously add in parting, that this Cyclopædia deserves in a high degree the encouragement of the profession, and especially of our young friends who are entering on its study.

II. Practical Anatomy of the Nerves and Vessels supplying the Head, Neck, and Chest: intended as a Guide for the Use of Students in the Dissection of those Structures. By Edw. Cock, Demonstrator of Anatomy at Guy's Hospital. 12mo, pp. 242. London, 1835.

The author considers it necessary to apologise for intruding another manual on the patience and the purse of students. He aspires to no more than to assist them in their dissections—to do by book, what, as a demonstrator, he has been in the habit of doing by words —to point out to the bewildered neophyte, that *this* is the nervus vagus, or *that* the lingual artery.

We agree with Mr. Cock that such labours, though humble, are useful in their place, and although the chief merits of a work of this description are simple accuracy, and easy method, those merits are not to be despised.

The student, conning a systematic work upon anatomy, is presented with the special description of each tissue, and of every set of vessels, separately, and in regular order. But dissection implies a very different process. Beginning at the superficies he passes to the centre, and finds many parts involved and intricate: nerves, arteries, and veins intersecting each other: and their numerous ramifications to be cleared and comprehended, before he can arrive at their parent trunks. This constitutes the real difficulty to the student in his earlier dissections, and this difficulty Mr. Cock attempts to diminish by the present manual. His plan is thus stated by himself.

" I have followed up the dissection of the head, neck, and chest, by dividing it into regional portions, each forming a distinct section of the work, commencing with the subcutaneous structures, and proceeding to those which are more deeply seated.

In carrying out this plan, I have constantly held one main object in view; that of rendering the description of the vessels and nerves, as continuous, and as little disjointed, as was compatible with the possibility of bringing them all successively under inspection: and without entering into prolix description or minute anatomical detail, I have endeavoured to afford an easy and efficient plan of dissection, and to enable the student to employ his time and labour to the greatest advantage.

It may be asked why I have limited myself to the description of vessels and nerves, excluding the bones and muscles, a knowledge of which is equally important to the surgeon. I answer, that I was unwilling to increase the size, and consequently add to the expense of this work, by treating of subjects which have been so ably handled by other authors, as to leave little room for improvement either in arrangement or description. I have only attempted to fill up a deficiency where I conceived it to exist, and to supply what I considered to be a desideratum to the dissector. How far I have succeeded my readers must decide; but experience renders me confident in the expectation, that, by the method and order recommended in this work, the student will have the advantage of seeing every important part contained in the head, neck, and chest, during the dissection of a single subject; and, though he may sometimes feel disappointed, at leaving for a time a nerve or vessel which he has traced through a certain portion of its course, he will in the end be amply repaid by the economy thus insured in the materials of his labor, and the opportunity afforded of studying each part in its natural position, in relation to surrounding structures."

We have glanced at the contents of the volume, and its execution seems to be respectable. The arrangement is not more complicated than necessary, and the descriptions are tolerably clear. No doubt it will assist both dissector and demonstrator, and form an acceptable companion to the student in his first attempts.

Manuals are by no means so popular as they were, even a few years ago. Both teachers and pupils have discovered that these short cuts to knowledge are apt to lead them a great way round. They have found that it is a farce, attempting to make minute anatomy very easy. It is a vast collection of facts, and it is as absurd to think of simplifying it very much, as to propose to facilitate in any great degree the acquisition of the multiplication table. The best manual of anatomy is that which does no more than convey its minutiæ in a natural order, and in perspicuous language. With those who are really anxious to become good anatomists, the work of M. Cloquet is, after all, more popular than any system or any manual that has yet been published. M. Cloquet's work might itself be simplified. The descriptions are at times involved, and frequently unnecessarily prolix. But their method and exactness more than counterbalance these disadvantages, and those who have once habituated themselves to M. Cloquet's style, are not likely to abandon his pages for any with which we have yet become acquainted.

III. COMPENDIUM OF THE LIGAMENTS; Illustrated by Wood-cuts, &c. with an Outline of the Dislocations, Fractures, Physiology, and Pathology. By A. M'NAB, jun. &c. 12mo, pp. 86.

This is a manual of a class which is nearly extinct—a manual which comprises anatomy, physiology, pathology and treatment. We doubt whether Mr. M'Nab is not too late in the field. However he may have satisfied himself of the necessity of such a production, we fear that he has yet to satisfy the public.

On turning over the pages, we see little to recommend. The descriptions are by no means good, and the wood-cuts are wretched. They are admirably calculated to confuse the student, both in what they attempt, and what they omit to shew. Mr. M'Nab's purse is likely to remain as empty as some of the bursæ mucosæ that he mentions.

IV. THE SURGEON'S PRACTICAL GUIDE IN DRESSING, AND IN THE METHODIC APPLICATION OF BANDAGES. Illustrated by numerous Engravings. By THOMAS CUTLER, M.D. Late Staff-Surgeon in the Belgian Army. 12mo. pp. 195.

The object of this unpretending, but really meritorious little work, is thus stated by the author in his preface.

"The work is divided into two parts: the first treating of Dressings and Bandages in general, and the principles of their application; the second, of Bandages in particular, classed according to the regions of the body, under the respective heads of Bandages of the Head and Neck, of the Trunk, of the Upper, and of the Lower Extremities. Each Bandage is moreover subdivided into its Composition, its Application, and its Use, particular observations being added as occasion may have required.

Throughout the whole work, which is illustrated in various parts by wood-cuts carefully executed by Messrs. Bagg and Son, the Author has endeavoured to render the details as brief as possible, without the sacrifice of either clearness or exactitude. He has drawn the materials necessary to its execution from practice, from personal observation in the British and Continental Hospitals, and from the works of the most distinguished writers upon Surgery generally, or upon this department in particular of the science; introducing nothing but what has appeared to him decidedly useful, he has still allowed for cases of great consequence an ample choice of means."

As a sample of what the contents of the volume are, we may select the heads of the matters treated of in the section on the Bandages of the Head and Neck. They are enumerated thus:—HEAD. 1, Head-band; 2, Triangular Bandage of the Head; 3, Four-Tailed Bandage of the Head; 4, Six-Tailed Bandage; 5, Capelina; 6, T Bandage of the Head; 7, Nodose Bandage.—FACE. 8, Double T Bandage of the Nose; 9, Another Bandage for the Nose; 10, Monocle; 11, Bandage for the Hare-lip Operation; 12, M. Thillaye's Bandage for the same; 13, Four-Tailed Bandage of the Chin; 14, Chevaster; 15, Bandage for Fracture of the Lower

Jaw, with Splint; 16, Mr. Lonsdale's Apparatus for the same.—Neck. 17, Bandage for Transverse Wounds of the Neck; 18, Bandage for Burns on the Fore Part of the Neck; 19, Bandage for Bleeding at the Jugular Vein; 20, Bandage for Wry-neck; 21, Professor Jörg's Apparatus for the same.

Our young friends may perceive how many things are told them, which they do not readily get in systematic works, nor, indeed, in lectures. We think Mr. Cutler's book deserving of encouragement on the part of pupils, and we strongly advise them to purchase it. There is a great deal of useful information in it—the woodcuts are numerous and intelligible—and the price of the work is moderate.*

V. A Series of Anatomical Plates. By Jones Quain, M.D. &c. First and Second Supplementary Fasciculi, and Fasciculi 30, 31, 32, and 33.

The supplementary fasciculi of this very deserving system of plates are dedicated to the display of the muscles, the division exhibiting the anatomy of which must now be considered as completed. On looking at it, we must admit that many of the lithographs are executed very cleverly, while some are inferior in point of clearness, and indeed of execution. These plates are, on the whole, much superior, as a system, to any that have been hitherto published in this country, and, taking their price and other circumstances into the account, they must be considered as a cheap and valuable acquisition to the anatomical student.

It is, perhaps, to be regretted that, whilst Mr. Quain was publishing a series of plates, he did not endeavour to render them more extensive and complete. With the utmost disposition to regard them with a favourable eye, they cannot be considered a national work—they cannot be put in competition with the plates of M. Bourgery, now in course of publication. We are convinced that a system of anatomical plates, on a much more extensive plan, and of a much more elaborate character than those before us, would be favourably received and widely patronized.

The four fasciculi, from 30 to 33 inclusive of them both, are appropriated to the delineation of the arteries. The thoracic and abdominal aorta, and the branches of the latter, are displayed. The drawings are very distinct, and the lithography equally clear.

We have often recommended the anatomical student to make himself the possessor of this work. As it proceeds, we may say, honestly, that our good opinion of it increases.

VI. The Clinique Medicale; or Reports of Medical Cases. By G. Andral, MD. &c. Condensed and translated, &c. by D. Spillan, M.D. &c. Part IV. containing Diseases of the Abdomen.

The present part of this valuable translation contains the diseases of the abdomen. It is essentially devoted to the pathology of fever.

M. Andral, after giving what may be called the history of fevers, considers, seriatim, the alterations found in the digestive tube—the circulatory apparatus—the respiratory apparatus—the apparatus of the secretions—the apparatus of the life of relation—and, finally, he offers some remarks on the different modes of treatment.

The present translation of M. Andral's valuable volumes is likely to prove very useful to practitioners in this country. The wide scope and philosophical exactness of the original work, rendered thus familiar to the body of the profession here, will tend to enlarge their views, and elevate them from the level of routine prescribers for symptoms, to which many are too apt to sink. We recommend Dr. Spillan's translation to the attention of our readers.

* The price is 6*s*. 6*d*.

VII. MANUEL DE MÉDECINE OPÉRATOIRE, &c. Par J. F. MALGAIGNE, Docteur en Médecine de la Faculté de Paris, &c. Paris, Baillière. 18mo. prix 6 francs.

This appears to us to be one of the most complete manuals of operative surgery that we have yet seen. It possesses a merit, which, for a manual of this description, is an useful one—it is portable, and not so frightfully voluminous as the work of M. Velpeau. The latter is well adapted for reference—this for use. Being the latest, it comprises the last proposals and inventions in the art of operating. It contains, too, chapters on the operations required for the teeth—on lithotrity—on chiropodism—on the operations required for the eye and ear.

From the very superficial glance we have been enabled to bestow upon the volume, it seems to be carefully and judiciously executed; and, on the whole, we should say that it is superior to any of the small works we have seen, and more convenient for use than the large.

